# Identifying Respiration-Related Aliasing Artifacts in the Rodent Resting-State fMRI

**DOI:** 10.3389/fnins.2018.00788

**Published:** 2018-11-02

**Authors:** Patricia Pais-Roldán, Bharat Biswal, Klaus Scheffler, Xin Yu

**Affiliations:** ^1^High-Field Magnetic Resonance Department, Max Planck Institute for Biological Cybernetics, Tuebingen, Germany; ^2^Graduate Training Centre of Neuroscience, International Max Planck Research School, University of Tuebingen, Tuebingen, Germany; ^3^Department of Biomedical Engineering, New Jersey Institute of Technology, Newark, NJ, United States; ^4^Department for Biomedical Magnetic Resonance, University of Tuebingen, Tuebingen, Germany; ^5^Athinoula A. Martinos Center for Biomedical Imaging, Massachusetts General Hospital and Harvard Medical School, Charlestown, MA, United States

**Keywords:** rat fMRI, physiological noise, EPI, ventilation rate, repetition time, resting state networks

## Abstract

Resting-state functional magnetic resonance imaging (rs-fMRI) combined with optogenetics and electrophysiological/calcium recordings in animal models is becoming a popular platform to investigate brain dynamics under specific neurological states. Physiological noise originating from the cardiac and respiration signal is the dominant interference in human rs-fMRI and extensive efforts have been made to reduce these artifacts from the human data. In animal fMRI studies, physiological noise sources including the respiratory and cardiorespiratory artifacts to the rs-fMRI signal fluctuation have typically been less investigated. In this article, we demonstrate evidence of aliasing effects into the low-frequency rs-fMRI signal fluctuation mainly due to respiration-induced B0 offsets in anesthetized rats. This aliased signal was examined by systematically altering the fMRI sampling rate, i.e., the time of repetition (TR), in free-breathing conditions and by adjusting the rate of ventilation. Anesthetized rats under ventilation showed a significantly narrower frequency bandwidth of the aliasing effect than free-breathing animals. It was found that the aliasing effect could be further reduced in ventilated animals with a muscle relaxant. This work elucidates the respiration-related aliasing effects on the rs-fMRI signal fluctuation from anesthetized rats, indicating non-negligible physiological noise needed to be taken care of in both awake and anesthetized animal rs-fMRI studies.

## Introduction

Low-frequency (< 0.1 Hz) fMRI signal fluctuation (LFF) related to spontaneous brain dynamic signaling has been observed using a number of different brain imaging modalities (Biswal et al., 1995; Obrig et al., 2000). Applying a series of data analysis including correlation, coherence and independent component analysis of LFF during resting state scans has revealed distinct “resting-state” networks (RSN) that represent potential functional connectivity across regions in the brain (De Luca et al., 2005; Fox et al., 2009). The “default mode” network (DMN) is one of the RSNs that has been reliably identified from resting-state fMRI (rs-fMRI) in the human brain (Raichle et al., 2001; Greicius et al., 2003). Similar DMN spatial correlation patterns have been detected in both anesthetized monkeys and rodents, using fMRI (Vincent et al., 2007; Mantini et al., 2011; Lu et al., 2012; Cabral et al., 2014; Stafford et al., 2014; Paasonen et al., 2018). This has allowed translational studies to specify the potential neuronal basis/underpins of the RSNs detected by fMRI in animal models. Animal fMRI has played a critical role in mapping the brain dynamics across multiple scales (Logothetis, 2002; Airaksinen et al., 2010; Hyder and Rothman, 2010; Mishra et al., 2011; Pan et al., 2011; Schulz et al., 2012; Yu et al., 2016; Yu, 2017; Albers et al., 2018; Wang et al., 2018). Multi-modal animal fMRI platform has been developed by merging fMRI with optogenetics (Lee et al., 2010; Liang et al., 2015; Yu et al., 2016; Albers et al., 2018), for cell/circuit specific activation, and with concurrent electrophysiological recordings (Goense and Logothetis, 2008; Shmuel and Leopold, 2008; Scholvinck et al., 2010; Pan et al., 2013). Also, fMRI brain mapping has been performed with parallel fiber optic measurements of the brain dynamic signals through genetically encoded sensors (e.g., detection of calcium with GCaMP or even of glutamate with GluSnRf) for identification of the LFF neural correlates (Schulz et al., 2012; Xie et al., 2016; Schwalm et al., 2017; Albers et al., 2018; He et al., 2018; Jiang et al., 2018; Wang et al., 2018). Thus, the multi-modal animal fMRI offers the possibility of studying the multi-site neural components underlying the RSNs.

Extensive efforts have been made to elucidate the confounding issues of LFF in the human brain, especially in the correction of motion artifacts from varied sources (Biswal et al., 1996; Welvaert and Rosseel, 2012; Murphy et al., 2013; Chen et al., 2015a; Jorge et al., 2015; Bright and Murphy, 2017). Besides the head motion-caused artifacts (Yang et al., 2005), the cardiorespiratory (CR) interference, e.g., the respiration-induced B0 field offset and cardiac pulsation etc., are the most dominant physiological noise source to a broad frequency range of the fMRI signal fluctuation (Hu et al., 1995; Noll et al., 1998; Van de Moortele et al., 2002; Razavi et al., 2008; Birn et al., 2008a; Starck et al., 2010; Birn, 2012; Murphy et al., 2013; Cordes et al., 2014). The CR-relevant parameters including arteriole CO2 (Wise et al., 2004), respiratory volume per time (RVT) (Birn et al., 2006, 2008b), and heart rate variability (HRV) (Shmueli et al., 2007), oscillate at frequencies < 0.1 Hz and directly interfere with the rs-fMRI signal correlation features. In addition, the slow sampling rate of fMRI could alias the high-frequency oscillation of the cardiac signal, and even the respiration artifacts at TR > 2 s, which can contaminate the low frequency range in human rs-fMRI studies (Biswal et al., 1996; Lowe et al., 1998; Noll et al., 1998; Dagli et al., 1999; Frank et al., 2001; De Luca et al., 2006). To solve the aliasing problem, high sampling rate imaging schemes or fMRI with specific TRs are needed to avoid the aliased oscillatory signals in the < 0.1 Hz low frequency range (Frank et al., 2001; De Luca et al., 2006; Posse et al., 2013; Cordes et al., 2014; Tong et al., 2014; Reynaud et al., 2017). Advanced imaging schemes and signal denoising methods have been developed to reduce these artifacts in human rs-fMRI studies (Biswal et al., 1996; Kiviniemi et al., 2003, 2005; Feinberg et al., 2010; Murphy et al., 2013; Feinberg and Setsompop, 2013; Liu, 2016; Abreu et al., 2017; Bright and Murphy, 2017; Caballero-Gaudes and Reynolds, 2017).

The CR-relevant confounding issues have been less concerned in animal rs-fMRI studies, especially in anesthetized animals with head-fixation to remove a large portion of the motion-relevant artifacts that are usually observed in awake human subjects (Kannurpatti et al., 2008; Zhao et al., 2008; Biswal and Kannurpatti, 2009; Pawela et al., 2010; Williams et al., 2010; Majeed et al., 2011). The respiratory rates of animals at different anesthesia vary largely, which can alter the respiration-related neck/chest motion patterns across different cases (Zhao et al., 2008; Williams et al., 2010; Bukhari et al., 2017). In many rs-fMRI studies using 2D-EPI sequences with different TRs (0.1 s -single slice- to 2 s), the CR-relevant aliasing effect was either reported negligible, or could be filtered out from the < 0.1 Hz LFF (Zhao et al., 2008; Biswal and Kannurpatti, 2009; Williams et al., 2010). Under the same anesthesia (e.g., medetomidine), the respiration-relevant motion artifacts have been reported to have different spatial patterns by different studies, either located only at the brain edge voxels using 300 ms TR (Magnuson et al., 2015), or to spread through the whole brain structure with severe aliasing interference at 3 s TR (Kalthoff et al., 2011). To better interpret the RSNs in animal rs-fMRI studies, the potential aliasing effect needs to be characterized carefully to avoid confounding the < 0.1 Hz LFF (Kalthoff et al., 2011). This is particularly critical for multi-modal fMRI studies that adopt 3D-EPI sequences to achieve even higher spatiotemporal resolution, so that more refined functional patterns from smaller brain nuclei can be matched with other imaging modalities in multi-scales (e.g., Wang et al., 2018) or even in awake animal fMRI studies (Ferenczi et al., 2016). Thus, for the implementation of a multi-modal fMRI methodology, a quality control to prevent or correct the physiological-related noise in the animal rs-fMRI signal is crucial to reliably investigate the neural basis of the RSNs detected in the animal brain.

In this study, we focused on deciphering the confounding rs-fMRI signal oscillation due to the respiration-related artifacts in anesthetized rats freely breathing or under ventilation with or without a muscle relaxant. By systematically varying the TR and the ventilation rates during the rs-fMRI acquisition, we characterized the aliasing effects, showing altered interference at different conditions. In addition, the frequency-specific power map demonstrated the voxel-specific sensitivity to the respiration-related motion artifacts with unique spatiotemporal dynamic interference. Because the ventilation mechanism enforces sinusoidal signals at very specific frequencies, it helps narrow the broad respiratory bandwidth of the free-breathing conditions, providing an efficient solution to avoid the overlap of the aliased oscillation to the < 0.1 Hz fMRI signal fluctuation. Furthermore, the muscle relaxant can significantly reduce the respiratory-induced B0 distortion and its corresponding aliasing artifacts to the LFF. In summary, this work provides a detailed view on aliasing effects of the rs-fMRI signal fluctuation from anesthetized rats, indicating a crucial CR interference issue needed to be taken care of in both awake and anesthetized animal rs-fMRI studies.

## Materials and Methods

### Simulations of the Physiological Noise Sampling at Different TR for fMRI

In order to simulate the aliasing effect of the respiratory motion in the fMRI signal acquired at a certain TR, a cosine wave oscillating at frequencies near the common rat ventilatory rate was created using Matlab (Mathworks, Natick, MA, United States). The generated signal was then down-sampled at frequencies equivalent to potential volume acquisition times (TR). The resulting waves were plotted to illustrate the effect of sampling an intruder ventilatory-driven signal at a certain TR.

### Mathematical Explanation of the Observed Aliasing Effects

The function *f*(*TR*) calculates the frequency *freqS* of a signal *S* that results from sampling an original signal *S*0 at a given *TR*.

$$f(TR)=abs(freqS0-k^{*}\frac{1}{TR})=freqS$$

with *k* = 1,2,3 … .

Supplementary Figure S1B shows an example of how a signal (e.g., motion from breathing at 60.1 breaths per minute) can be aliased by using typical TRs (i.e., TR > 0.5 s). When using a sampling frequency equal to the peak frequency S0, the resampled signal (S) is flat (frequency = 0) (see Supplementary Figure S1A middle inferior panel). In the practice, complete cancelation of the signal is often not possible, when dealing with analog devices, but by getting very close to the peak frequency, an extremely slow aliased signal can remain, which would lie out of the rs-fMRI analysis (freqS < < < 0.01 Hz, Supplementary Figure S1B). By knowing the specific rate at which the animals breathe (e.g., by imposing a respiration rate with ventilatory support) it is possible to choose an appropriate TR that minimizes the interference of the aliased signal with the spectrum of interest (e.g., 0.01–0.1 Hz).

### Animal Subjects, Procedures, and Experimental Design

In order to test whether aliasing occurs in the fMRI signal upon spontaneous breathing, nine adult (300–400 g) male Sprague Dawley rats were included in the study and their fMRI signal was assessed under anesthesia at three different conditions: (1) spontaneous breathing through a mask; (2) ventilated, not paralyzed; and (3) ventilated and paralyzed. All animal procedures were approved by the Animal Protection Committee of Tübingen (Regierungspräsidium Tübingen).

All animals were initially anesthetized with 5% isoflurane in chamber. In order to track changes in the blood pressure, the femoral artery was catheterized and connected to a blood pressure transducer (Biopac, Goleta, CA, United States). As in further steps the effect of a muscle relaxant was to be assessed, the femoral vein was also catheterized and connected to an infusion pump. The inner ear cavity was filled with paste to avoid air-tissue interfaces near the brain. A closed-loop heating system was used to keep the rectal temperature at 37°C during the entire experiment, ensuring stable physiological conditions. The end-tidal CO2 was also controlled throughout the experiment using a Respironics-Novametric monitor. A chest transducer was placed under the chest of the animal and constantly used to monitor breathing through a Biopac acquisition system, at 5000 samples per second.

During acquisition of the fMRI, 6 animals were kept anesthetized with 1.5–2% isoflurane, 1 animal was anesthetized with alpha-chloralose (25 mg/Kg⋅h), 1 with urethane (1 dose of 1–2 g/Kg) and 1 with medetomidine (0.05–0.5 mg/Kg⋅h), to rule out the contribution of a specific anesthetic to the observed effects. First, the not-yet-intubated animals were transferred to the 12 cm horizontal bore of a 14T MRI scanner and breathed oxygen-enhanced air (∼30% oxygen) through a mask. In the second part of the study, animals were intubated and maintained at a constant ventilatory rate that could be varied between scans. A last study was carried out in animals anesthetized with isoflurane with concurrent intravenous infusion (∼1–2 mg/Kg⋅h) of the muscle paralyzer agent Pancuronium (typical fMRI setup). All animals were euthanized right after the fMRI study based on the termination procedure from the approved protocols.

### fMRI Acquisition

To detect the BOLD signal in the anesthetized animals, 2D and 3D Echo Planar Imaging (EPI) sequences were used, covering a field of view (FOV) of 2.24 cm × 1.92 cm × 1.92 cm with a matrix size of 56 × 48 × 32, achieving a resolution of 400 μm × 400 μm ×600 μm (all the data shown in the main figures correspond to 3D EPI, unless stated otherwise -Supplementary Figure S7 is an example of a 2D EPI-). In order to determine whether different TRs modified the frequency of the aliased signal, the TR was varied between scans (TE was kept at 9.75 ms). A phantom containing Phosphate Buffered Saline (PBS) was located over the animal’s head to compare the signal detected inside the brain with a signal experiencing similar motion in an inert tissue. A surface coil of diameter 2.2 cm was used to transmit and receive the RF signals. For anatomical registration purposes, RARE images were also acquired using a TR of 4000 ms, TE of 9.025 ms, FOV of 2.24 × 1.92 × 1.92 and matrix size of 128x128x32 (resolution of 175 μm × 150 μm × 600 μm). Table 1 summarizes the conditions and fMRI parameters used in our study.

**Table 1:** Experimental conditions.

TR (s)	No V	V-54	V-56	V-58	V-59	V-60	V-61	V-62	V-64	V-66
0.5	wo P					w & o P				
0.8	wo P					w & o P				
0.9	wo P					w & o P				
1	wo P	wo P	wo P	wo P	wo P	w & o P	w & o P	w & o P	w & o P	w & o P
1.1	wo P					w & o P				
1.2	wo P					w & o P				
1.5	wo P					w & o P				
2	wo P					w & o P				


*The table summarizes the parameters used in our study in terms of ventilatory frequency (in beats per minute), TR, and the use of a muscle relaxant (Pancuronium). No V, not ventilated (using mask); V-N, Ventilated (at the rate N, in breaths per minute); P, Pancuronium (wo: without, w & o, with and without).*

### Analysis of the Data

The purpose of the analysis was to determine the influence of the respiration in the fMRI time course. Therefore, both signals (i.e., the chest–derived motion trace and the fMRI time-course), were considered for analysis.

### Assessment of the Spatial Distribution of the Interference

The amplitude of the fluctuations in the BOLD signal was considered as an indicator of the level of contamination in the data. In order to detect the brain areas most vulnerable to the respiration-related interference, a power map was created in AFNI (Cox, 1996), by calculating the BOLD signal change at each voxel. The function 3dPeriodogram was used to obtain 1024 frequency components and their corresponding power in each functional scan. 3dTstat was used to average the power of the frequencies in the range of 0.005 to 0.4 Hz in each voxel, resulting in a color-coded power map. In order to compare different animals and to average maps together, all scans were registered to a template using 3dAllineate. First, the anatomical datasets were aligned, and later the registration matrix was applied to the functional scans.

### Comparative Analysis Between Chest Motion and fMRI Signal

In order to compare the respiratory-driven chest motion and the fMRI time-course, both signals were plotted in temporal and frequency domains. fMRI time courses were extracted by manually drawing a mask of 4 voxels from inside the brain and from a phantom placed over the animal’s head. The AFNI function 3dMaskave was used to compute the average of the 4 voxels and output a 1D file. To extract single voxel time courses, the AFNI function 3dmaskdump was implemented. The time courses were then read in matlab using dlmread. In order to create a signal that simulates the chest motion detected from the fMRI, chest movement traces were down-sampled to 1/TR for each fMRI acquisition and included in the analysis. The fast Fourier transform (fft) function of matlab was used to calculate the power spectral density (PSD) from the original chest movement trace, from the down-sampled chest trace, and from the fMRI time courses. The full width at half maximum (FWHF) of the peak aliased frequency was calculated on the enveloped chest and fMRI spectra using matlab, to assess their similarity in terms of bandwidth and differentiate between mask and ventilation conditions. A two-tailed not-paired *t*-test was performed to assess the differences between groups. Errors in figures represent standard error of the mean.

### Analysis of the Variance

To quantify the degree of variability that is incorporated to a group of scans solely based on the aliasing effect (i.e., variability of the fMRI signal upon using different ventilatory rates or TRs), spectral signal similarity was evaluated for each pair of conditions (e.g., time course acquired at TR = 1 vs. TR = 1.2 s, or time course acquired from the animal ventilated at 60 bpm vs. 61 bpm) by using the corr2 function in matlab applied to the PSDs. Correlation matrices were built to visualize the effect across all different conditions. Signal amplitude (i.e., BOLD signal change) was calculated as the standard deviation within the time course. A two-tailed not-paired *t*-test was performed to assess the difference in averaged signal amplitude (indicative of signal contamination) between fMRI time courses from spontaneously breathing animals vs. paralyzed animals. Errors in figures represent standard error of the mean.

### Power Spectrum Characterization

In order to have a measure of the spectral specificity of the respiration-induced interference at different conditions (i.e., how wide the contaminated frequency range is or how much it spreads from a center frequency in an animal breathing through mask or mechanically ventilated), a map showing the fractional amplitude of low frequency fluctuations (fALFF) at specific frequency bands was generated using the function 3dRSFC (Zou et al., 2008), to cover a bandwidth of 0.01 Hz at the peak aliased frequency and the immediate lower and higher spectral components. To further verify the effect of ventilating the animals on the width of the frequency range subjected to respiratory-driven interference, the matlab function bandpower was used to calculate the average power within a 0.01 Hz bandwidth centered at the peak aliased frequency or the immediate superior and inferior neighbors. A two-tailed paired *t*-test was performed to assess the difference in power between the peak and the neighbor frequencies. Errors in figures represent standard error of the mean.

Table 2 provides the *p*-values obtained from the different *t*-tests performed during analysis.

**Table 2:** *p*-values.

Figure 2E	Mask chest	Mask brain	Vent,noP chest	Vent,no P brain		
Mask chest		0.70	2.52^∗^10^∧^-8			
Mask brain	0.70			7.00^∗^10^∧^-6		
Vent, noP chest	2.52^∗^10^∧^-8			0.015		
Vent, no P brain		7.00^∗^10^∧^-6	0.015			

Figure 2F	**Mask-peak**	**Mask-neigh**	**Vent,noP-peak**	**Vent,noP-neigh**	**Vent,P-peak**	**Vent,P-neigh**

Mask- Peak		2.5^∗^10^∧^-2	0.90		4.02^∗^10^∧^-12	
Mask- neigh	2.5^∗^10^∧^-2			9.68^∗^10^∧^-11		2.83^∗^10ˆ-14
Vent,noP-peak	0.90			8.91^∗^10^∧^-4	2.97^∗^10^∧^-4	
Vent,noP-neigh		9.68^∗^10^∧^-11	8.91^∗^10^∧^-4			1.10^∗^10^∗^-3
Vent,P-peak	4.02^∗^10^∧^-12		2.97^∗^10^∧^-4			4.40^∗^10^∧^-4
Vent,P-neigh		2.83^∗^10^∧^-14		1.10^∗^10^∗^-3	4.40^∗^10^∧^-4	

Figure 5D	**noP**	**P**				

noP		1.13^∗^10^∧^-5				
P	1.13^∗^10^∧^-5					


*The table summarizes the *p*-values obtained from the *t*-tests performed during analysis, organized by figures.*

## Results

### Identification of the Aliased Respiration-Driven Artifact in Anesthetized Rats

**FIGURE 1:**
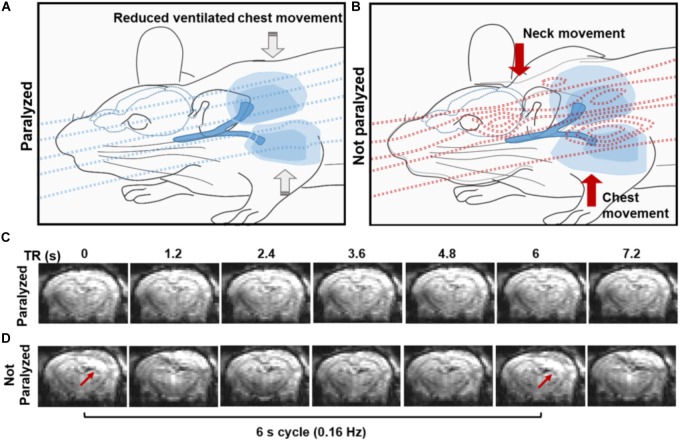
Effect of the spontaneous respiration on the B0 field. **(A,B)** The schemes illustrate the B0 field at the iso-center of the MR bore in the presence of a paralyzed **(A)** or a non-paralyzed animal **(B)**. Note how the B0 is relatively homogeneous when a paralyzed animal is passively ventilated due to the lack of large body motion and how B0 inhomogeneities emerge upon active breathing involving diaphragm, intercostal and neck muscle movement during the respiration **(B)**. **(C,D)** Coronal sections from a 3D EPI sequence acquired at 1.2 s TR, showing the effect of the B0 (in)homogeneity in a paralyzed **(C)** and spontaneously breathing animal **(D)**. Note the distortions in the images acquired from the non-paralyzed animal, compared to the paralyzed condition.

A 3D EPI-fMRI method was used to study the potential interference between the animal respiration and the rs-fMRI signal in anesthetized rats. Figure 1 illustrates the potential disruption of B0 field homogeneity due to the respiration-induced movement of the chest/neck, which results in distorted EPI images with characteristic periodic patterns in ventilated rats without muscle relaxant. It is worth noting that the periodic motion artifacts can be detected in animals under different anesthesia (e.g., medetomidine, urethane, isoflurane, and alpha-chloralose) (Supplementary Figure S2). Since muscle relaxants avoid the movement of the chest/neck, they reduce the respiration-induced B0 offset during fMRI acquisition, therefore, 3D EPI images show little distortion in paralyzed rats under ventilation (Figure 1C).

**FIGURE 2:**
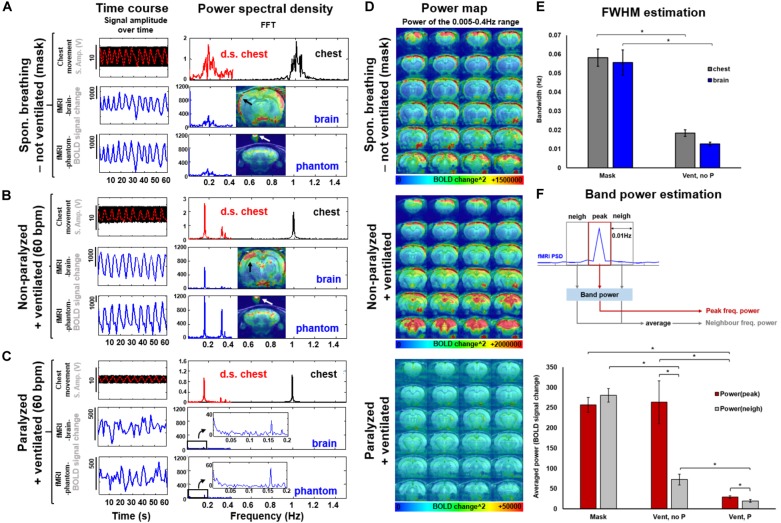
Spectral and spatial characterization of the respiratory interference at 3 conditions: spontaneously breathing through a mask, ventilated and ventilated while paralyzed. **(A,B,C)** The original chest movement recorded at a sampling rate of 5000 is shown as a black trace. The down-sampled chest-motion (resampled at 1/TR) is shown in red. Note the smaller amplitude of this signal in the paralyzed condition **(C**, vs. **A** or **B)**. The fMRI time course of a voxel in the brain or in a phantom (over the animal’s head) is shown in blue. The frequency decomposition of the signals is shown on the right. Note the similarity between the spectra of the aliased chest and fMRI signal. **(D)** The power maps show the distribution of the interference for each condition (averaged from 5 different animals). Note the low power scale in paralyzed animals. **(E)** The graph shows the spectral FWHM (i.e., spreading) of the peak breathing frequency and the aliased signal in the fMRI time course for non-intubated (mask, *N* = 26) and ventilated non-paralyzed animals (*N* = 17). **(F)** The graphs show the averaged power calculated within a frequency range that contains either the peak frequency (i.e. aliased from chest motion) or the limiting/neighbor frequency bands. *N*(Mask) = 16 trials from 4 animals, *N*(Vent,noP) = 16 trials from 3 animals, *N*(Vent,P) = 14 trials from 4 animals. Statistical test within conditions: two-tailed paired *t*-test. Statistical test between conditions: two-tailed not-paired *t*-test. (^∗^): *p*-value < 0.005.

To specify the aliasing effect that occurs when the respiratory cycle is sampled at a given TR (see simulated data in Supplementary Figure S1), the respiratory signal (i.e., the chest movement) was recorded simultaneously with the rs-fMRI signal in anesthetized rats under three conditions: (1) not-ventilated, spontaneously breathing through mask, (2) ventilated, without muscle relaxant (i.e., not paralyzed), and (3) ventilated, with muscle relaxant (i.e., paralyzed). In Figure 2, the TR of the 3D EPI-fMRI was 1.2 s, i.e., 0.83 Hz. As shown in the PSD of the respiratory signal, the freely breathing rat (i.e., not ventilated -breathing through mask-) exhibits a broad frequency peak centered at ∼1 Hz (“chest”, Figure 2A). The FWHM of this broad peak in non-ventilated rats is ∼0.06 Hz ± 0.004 Hz (Figure 2E). The aliasing effect of this respiratory signal at a sampling rate of 0.83 Hz causes a slow oscillatory signal at the 0.17 Hz ± 0.03 Hz, which is observed in both, brain voxels and phantom voxels positioned at the top of the rat head (Figure 2A). A strong correlation between the peak frequency observed from the aliased (i.e., down-sampled) chest motion and the fMRI time course of both, brain and phantom voxels, suggests that the artifacts originate from the respiratory-induced B0 offset and not from brain functional dynamic signals. Similarly, the fMRI signal from rats ventilated at 1 Hz shows clear aliasing effect at 0.17 Hz, but the bandwidth of the aliased signal, assessed as the FWHM of the aliased peak, is much sharper than the observed in not-ventilated animals (Figure 2E, *p*-value = 7^∗^10^∧^-6), in both brain and phantom voxels (Figure 2B). The aliased signal is significantly reduced in paralyzed ventilated rats (Figures 2C,F).

### Prevalence and Characteristics of the Aliased Signal Across Different Ventilatory Conditions

To specify the spatial distribution of the respiration-related motion artifacts on the 3D EPI images, the mean voxel-wise power map (0.005–0.4 Hz) was calculated for each group (Figure 2D), showing that dorsal cortical areas and ventral brain regions are more sensitive to the motion artifacts. However, no generalized spatial patterns of motion artifacts can be achieved; instead, the respiratory-related motion and, hence, the interferential signal, varies across animals (Supplementary Figure S3). The significantly lower power estimate at the peak frequency in the paralyzed group (*p*-value = 2.9^∗^10^∧^-4, compared to the non-paralyzed ventilated condition) suggests a reduced aliasing effect in animals without spontaneous breathing (Figure 2D, bottom map, and Figure 2F). Figure 2F and Supplementary Figure S4 demonstrate the differences in power between the aliased center frequency and the collateral frequency bands at three conditions (mask, ventilated and ventilated paralyzed). Figure 2F shows the averaged power within a bandwidth of 0.01 Hz calculated at the peak and the neighbor frequencies across different animals, showing a significantly sharper effect (i.e., less contribution of the neighbor frequencies) in ventilated animals, compared to freely breathing rats (*p*-value = 9.68^∗^10^∧^-11). Supplementary Figure S4 provides the fALFF maps specific for the peak and neighbor frequency bands in a representative animal. The power estimates in the adjacent frequency bands are much lower when the animal is ventilated, in comparison to the free-breathing condition. This indicates that ventilation can prevent the spread of respiratory-motion aliasing effects along a broader frequency bandwidth (Figures 2E,F and Supplementary Figure S4). This is particularly important when the conventional TR at 1s is used, which causes the aliasing oscillatory effect at < 0.1 Hz to directly interfere with the low-frequency rs-fMRI signal fluctuation (Supplementary Figure S5). This result demonstrates that ventilation can be used to confine the aliasing oscillatory effect to a sharp frequency range.

### Characterization of the Spatial and Temporal Patterns of the Respiration-Driven Aliasing Effects on rs-fMRI: Influence of the TR and the Ventilatory Rate

**FIGURE 3:**
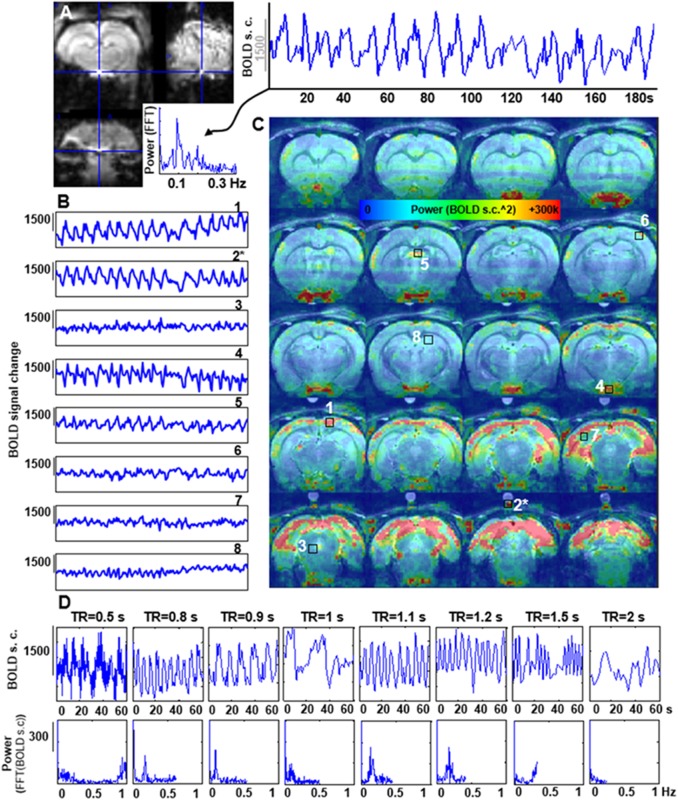
Artifactual slow fMRI waves and TR dependency. **(A)** Location of the extracted voxel, fMRI time course and PSD from a 3D EPI acquired in an animal breathing spontaneously through a mask, showing an artifactual slow wave with peak frequency of ∼0.09 Hz (TR = 0.9 s). **(B)** The graphs show 180 s of fMRI time course from 8 different voxels in a spontaneously breathing animal (TR = 0.9 s). Numbers indicate the location of the voxels (see **C**). (^∗^) indicates a phantom voxel (registration is not perfect due to misalignment between the anatomical and functional scan). **(C)** The map shows the distribution of the power of frequencies within the range 0.005–0.4 Hz. **(D)** fMRI time courses acquired at eight different TRs in a spontaneously breathing animal (not ventilated). Note how the frequency of the artifactual oscillations changes according to the chosen TR.

**FIGURE 4:**
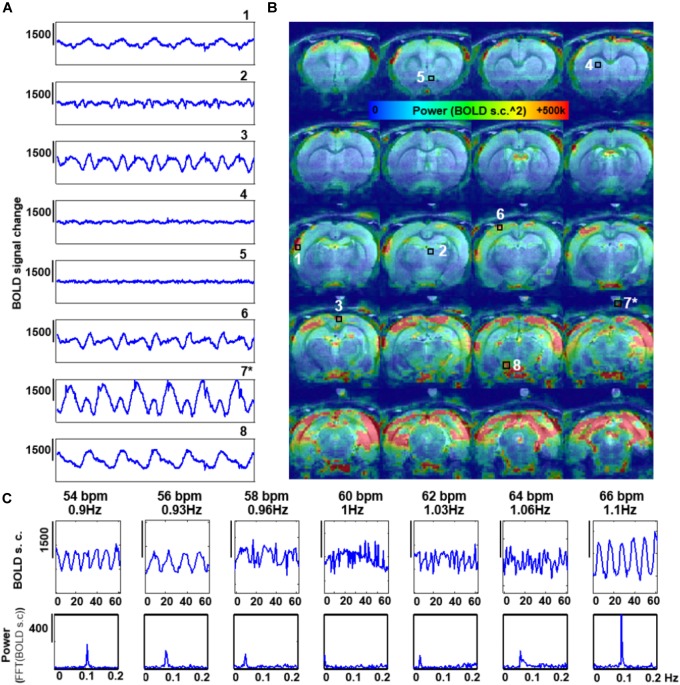
Effect of the rate of artificial ventilation on the fMRI time-course. **(A)** The graphs show 300 s of fMRI time course from 8 different voxels, extracted from a 3D EPI in an animal ventilated at 59 bpm (0.96 Hz) (TR = 1 s). Numbers indicate the location of the voxels (see C). (^∗^) indicates a phantom voxel (registration is not perfect due to misalignment between the anatomical and functional scan). **(B)** The map shows the distribution of the power of frequencies in the range 0.005–0.4 Hz. **(C)** fMRI time courses acquired with TR = 1s in a non-paralyzed animal ventilated at seven different rates.

The features of the aliased oscillation were evaluated in non-paralyzed animals at multiple voxels across different parts of the brain and a phantom located over the animal’s head. Different voxels exhibited different signal interference, in both, mask-breathing and non-paralyzed ventilated animals (Figures 3B,C, 4A,B), which demonstrates a highly varied effect of the respiration on the rat fMRI signal. In order to reduce the potential interference of the aliased respiration on the LFF (< 0.1 Hz), different TRs were used (0.5 s to 2 s), demonstrating that the aliasing oscillation can be shifted along the spectrum in non-ventilated animals (e.g., to be out of the < 0.1 Hz low-frequency range) (Figure 3D and Supplementary Figures S1B, S5). Similarly, to avoid the interference to the < 0.1 Hz rs-fMRI signal fluctuation, the ventilation rate could be altered in mechanically ventilated animals with fixed TR (1 s, Figure 4C and Supplementary Figure S1A), as an alternative strategy for anesthetized animal rs-fMRI studies without employing muscle relaxant agents.

### Examination of the Variability and Magnitude of the Aliasing Effect on the rs-fMRI in Ventilated Rats: Influence of Muscle Relaxants

**FIGURE 5:**
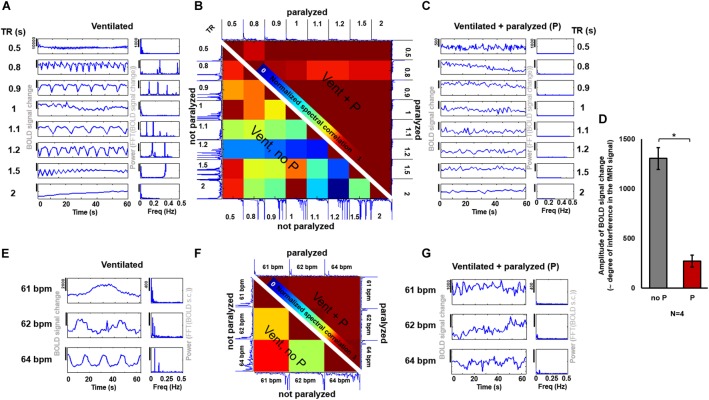
Effect of a muscle relaxant on the emergence of artefactual oscillations. **(A**,**C)** The graphs show the time courses and PSDs of the fMRI signal from an animal ventilated at 1 Hz, acquired at 8 different TRs, when the animal is not paralyzed **(A)** and when it is paralyzed with a muscle relaxant **(C)**. **(B)** The matrix shows the correlation values between the spectra from the different time courses acquired at different TRs (under the same ventilatory rate) in a spontaneously breathing animal (i.e., ventilated not paralyzed, as in “**A**”), shown on the lower left half of the matrix, and in a ventilated paralyzed animal (as in “**C**”), shown on the upper right half of the matrix (the matrix is not symmetric, instead it incorporates one half of the “non-paralyzed” matrix and one half of the “paralyzed” matrix, to avoid redundancy). High values indicate a higher correlation between time courses (i.e., low variability, as observed between time courses from paralyzed animals). **(D)** The bar plot shows the averaged amplitude of the BOLD signal change (indicative of the level of interference) in non-paralyzed ventilated animals and in paralyzed ventilated animals (*N* = 26 time courses, acquired at variable TRs, from 4 animals, for each of the two conditions). (P) Pancuronium. **(E**,**G)** The graphs show the time courses and PSDs of the fMRI signal from an animal ventilated at 3 different rates, acquired at TR = 1 s, with **(G)** and without **(E)** a paralyzer. **(F)** The matrix shows the correlation between the spectra of the signals acquired at different ventilatory rates (same TR) while the animal had spontaneous breath (i.e., ventilated not paralyzed, as in “**E**”), shown on the lower left half of the matrix, and of signals acquired while the animal was ventilated and paralyzed (as in “**G**”), shown on the upper right half of the matrix (the matrix is not symmetric, instead it incorporates one half of the “non-paralyzed” matrix and one half of the “paralyzed” matrix, to avoid redundancy). High values indicate a higher correlation between time courses (i.e., low variability). (^∗^): *p*-value < 0.005.

The fMRI signal is an indirect measure of neuronal activation and therefore, any variability introduced by external sources is undesired and should be minimized. In order to assess the degree of variability between data acquired under different ventilatory rates or different TRs (i.e., different aliasing effects), and to determine whether the use of a paralyzer agent could reduce the differences across scans, fMRI time courses were acquired at different sampling and ventilatory conditions with and without the muscle relaxant Pancuronium (Figure 5). In ventilated rats anesthetized with constant 2% isoflurane and without muscle relaxant, the fMRI time courses acquired at different TRs exhibited a large between-scan spectral variability, compared to groups of scans performed during infusion of the paralyzer agent (Figure 5B). Similarly, the spectral correlation between scans acquired at different ventilatory rates was lower in spontaneously breathing animals (Figure 5F). The magnitude of the aliased-motion artifact, estimated as the standard deviation within the fMRI time course, was calculated across animals, revealing a significantly lower contamination of the fMRI signal in the paralyzed condition (Figure 5E, *p*-value = 1.13^∗^10^∧^-5). These experiments demonstrate that the respiration-induced interference on the fMRI signal and the subsequent inter-scan variability can be ameliorated by providing a muscle relaxant during the fMRI study. In agreement with this, the seed-based correlation analysis of rs-fMRI acquired from ventilated rats without muscle relaxant (no denoising procedure) showed highly varied correlation patterns throughout the brain, in contrast to the more specific correlation pattern observed from rats ventilated and paralyzed with a muscle relaxant, which identifies RSNs more reliably (Supplementary Figure S6), as previously reported (Williams et al., 2010; Lu et al., 2012; Hsu et al., 2016; Bajic et al., 2017).

## Discussion

In this study, the aliasing effect due to respiration-induced B0 distortion was systematically characterized in the rs-fMRI of anesthetized rats at varied fMRI imaging and respiratory controlling conditions: different TRs, altered respiration rates, free-breathing vs. ventilation (with or without muscle relaxant). The ventilation scheme was found to significantly narrow the broad respiratory frequency bandwidth of the free-breathing anesthetized rats (from FWHM≅0.06 Hz to FWHM≅0.01 Hz) and reduce the potential aliasing interference on the < 0.1 Hz fMRI signal fluctuation, which can be further reduced by the muscle relaxant. The characterization of the spatial and temporal dynamic features of the aliasing effects due to B0 distortion from individual animals allows choosing appropriate TRs or ventilation rates to avoid the aliased oscillation within the 0.01–0.1 Hz range of the fMRI signal. This may be particularly useful in fMRI studies intended to study brain dynamics in awake animals, which are becoming a common platform to investigate neurological processes without the potential confounder effects from general anesthetics. Despite the benefit of being anesthetic-free, imaging the awake brain involves the need of coping with other sources of confusion variables like potential stress or spontaneous voluntary movement of the limbs or body and, inevitably, spontaneous breathing. While the stress and voluntary motion can be avoided by exhaustive training with the animal, the last one is a factor inherent to and inseparable of the awake condition. Therefore, faced the unfeasibility of applying a muscle relaxant to these animals, it is critical to understand the implications of imaging a spontaneously breathing rodent before acquiring rs-fMRI data and, if possible, choose adequate parameters that allow an easy discrimination between neurological and artifactual signals (e.g., deviating the aliased signal from the spectral range intended for analysis by choosing a convenient TR). Given the physiological noise of the rs-fMRI detected from anesthetized rats, this work suggests that the denoising methods developed for human fMRI (Hu et al., 1995; Glover et al., 2000; Birn et al., 2014; Caballero-Gaudes and Reynolds, 2017) should be adapted and implemented in the routine rs-fMRI animal studies.

### Divergence Between the Aliasing Interference in Humans and Rodents

Human cardiovascular and respiratory signals in the young adult fluctuate at 0.66–1.66 Hz and 0.16–0.33 Hz, respectively (Fleming et al., 2011). Human respiration can, therefore, be sampled if relatively short TRs are used (< 1.5 s), preventing aliasing. In contrast, the cardiovascular oscillations constitute a clear source of aliasing in human fMRI (TR needs to be ∼0.3 s to sample the human heart rate appropriately) (Hu et al., 1995; Kiviniemi et al., 2005; Feinberg et al., 2010; Hugger et al., 2011). Despite the similarity between the neurological signals of human and rodents (e.g., EEG spectrum), the heart and respiratory rates are approximately 5 times faster in the rat (5–8 Hz and 0.9–1.5 Hz, respectively), with their respiratory cycle matching the human cardiac cycle (Zhao et al., 2008; Williams et al., 2010). The spectral features of the rat cardio-respiratory signals imply the need of correction methods to avoid aliasing in the 0.01–0.1 Hz fMRI signal. Here, we have shown how the respiratory interference can be detected and shifted away from the rs-fMRI analysis by changing the ventilatory rate or the rate of fMRI sampling (i.e., TR). This is possible due to the fact that the aliased frequency depends on the frequency of the interference (i.e., the respiratory rate) and on the sampling rate (i.e., the TR), which is a consequence of the Nyquist Sampling Theorem, stating that if the rate of sampling is less than twice the signal frequency, aliasing occurs, with f_alias_ = | f_resp_-n^∗^f_sample_|, *n* = 1, 2, …, f_sample_ = 1/TR (Holst, 1996; Kiviniemi et al., 2005). Future studies using fast imaging schemes (e.g., line scanning with 50 ms TR Yu et al., 2014) could be used to characterize the cardiovascular contributions to the fMRI signal in rodent studies.

### 3D vs. 2D EPI fMRI Methods

In this work, we demonstrated the aliasing effect of the respiratory-driven artifacts in the rat rs-fMRI acquired as 3D EPI, which is a highly efficient sequence to acquire whole-brain fMRI signal. Nevertheless, a significant portion of the animal and human fMRI studies are performed with multi-slice (2D) EPI (Keilholz et al., 2004; Bruno et al., 2010; Magnuson et al., 2010; Williams et al., 2010; Lu et al., 2012; D’Arceuil et al., 2013; Chen et al., 2015b), which is less sensitive to physiological noise than its 3D counterpart (Lutti et al., 2013). While 2D EPI slices are acquired consecutively within ∼30 ms, with each slice sensing a different dynamic B0 field during a small portion of the respiratory cycle, 3D EPI schemes sample the whole brain slab in an additional phase encoding step, which incorporates the B0 field distortion through the whole respiratory cycle. This difference will certainly make the 3D EPI method more sensitive to the B0 field distortions due to respiration. To account for the potential differences between both imaging schemes, multi-slice 2D EPI was acquired in animals to demonstrate the presence of a similar aliased respiratory signal in both rs-fMRI paradigms (Supplementary Figure S7). Although the abovementioned aliased signals could be detected in both imaging schemes, the aliased signal interference remains but is reduced in the 2D EPI method in comparison to the 3D EPI [a more exhaustive comparison between the sensitivity of both methodologies is beyond the purpose of the current study, for a discussion on the topic see: (Goerke et al., 2005)]. While physiological noise can be regressed out retrospectively from 2D EPI datasets by slice-selective correction methods (Glover et al., 2000; Hutton et al., 2011), other strategies, e.g., ICA-based artifact removal (Griffanti et al., 2014), may be applied to 3D EPIs to diminish the noise generated by the aliased cardiorespiratory interference (Caballero-Gaudes and Reynolds, 2017).

### Complexity of the Respiration-Induced B0 Distortion Aliased Signal Throughout the Brain

The B0 offset induced by respiration-related motion causes dynamic B0 spatial distortion through the respiratory phasic cycles. Figures 2D, 3C, 4B show how the oscillatory signals due to motion-related artifacts dominate the fMRI signal fluctuation of the cerebellum, cortex and hypothalamic area across a frequency bandwidth (0.005–0.4Hz). Despite a major susceptibility of the dorsal, occipital and ventral regions, the distribution of the artifactual signals can vary between animals with different respiratory motion (Supplementary Figure S3), which shows the challenges of the generalized correction method. Paralysis of the ventilated animal resulted in a significantly reduced interference, as passive ventilation does not involve recruitment of accessory muscles in the chest or neck of the animal, which are the major contributors to the B0 inhomogeneities (Raj et al., 2001; Van de Moortele et al., 2002). However, other physiological variables not coupled to motion, such as the arterial CO2 signal (Wise et al., 2004), can change with each respiratory cycle and its direct vascular interference can be aliased into the rs-fMRI signal, even in paralyzed animals. The aliasing of these signals would cause a strong correlation between large vessels (Dagli et al., 1999). Besides the breathing-related physiological variables, an enveloped blood pressure signal of frequency ∼0.01–0.04 Hz (Meyer waves, dependent on sympathetic activity) may also appear and contaminate the BOLD signal near large vessels (Diehl et al., 1995; Pfurtscheller et al., 2017). Importantly these signals would not be a consequence of aliasing (e.g., generated due to insufficient sampling) but may be caused by real cardiac-induced slow fluctuations (Bhattacharyya and Lowe, 2004). The ventral area (i.e., near the circle of Willis) highlighted in the power map of Figures 3, 4 may also potentially indicate a contribution of an aliased cardiorespiratory signal in the rs-fMRI data.

### Spontaneous vs. Imposed Ventilation

Free-breathing subjects may exhibit a broad respiratory frequency bandwidth (FWHM≅0.06 Hz) along the acquisition time (Figures 2A,E and Supplementary Figures S5, S7, black and red traces of the mask condition). This produces an equally broad bandwidth of frequencies aliased into the fMRI signal (Figures 2A,E and Supplementary Figures S5, S7, blue traces of the mask condition). In 2D EPI, different slices within the volume may be acquired at different phases of the varied respiratory cycles along the scan time, which leads to mild aliasing periodic patterns that could be diminished with slice-selective denoising methods (Glover et al., 2000; Hutton et al., 2011). In 3D EPI, the B0-offset throughout the varied respiratory cycles may produce artifacts along the second phase encoding direction that is harder to be corrected using the existing regression methods. Ventilation of animals at constant rates during scans forces the respiratory cycle to remain unvaried, therefore avoiding phase difference between cycles and sharpening the spectrum of the respiratory-derived motion within functional scans (Figure 2E and Supplementary Figure 4b). By providing ventilation at specific rates, the process of identification of the aliased frequency is optimized, allowing to exclude the given spectral components with band-stop filters. Furthermore, paralyzer agents could be used to further dampen the respiratory interference.

## Conclusion

Here, we present evidence for an interference in the rs-fMRI signal fluctuation from the respiration-related motion in animals breathing through mask or artificially ventilated, which can be observed as waves within a large frequency range depending on the length of the particular respiratory cycle and on the TR used to sample the fMRI signal (i.e., aliasing effect). The artifacts can be significantly dampened by using muscle paralyzers like Pancuronium for anesthesied animals of terminal studies. For the chronic or longitudinal studies, post-processing methods with retrospective regression (Hu et al., 1995; Glover et al., 2000), e.g., RETROICOR, can be used to attenuate the respiration-induced artifacts, as has been routinely used for human fMRI studies. Additionally, the use of a convenient TR may further simplify the cleaning process by shifting the aliased respiratory-driven artifacts to a specific frequency range that can be filtered out from the analysis. Our observations suggest that recording of respiratory cycles and blood pressure in parallel to fMRI and data cleaning (i.e., removal of physiological noise) constitutes a necessary step in small animal fMRI as well, especially in awake or non-paralyzed anesthetized rodent studies.

## Author Contributions

XY led the research. XY, KS, and PP-R designed the experiments. PP-R and XY performed the experiments. PP-R XY and BB guided the analysis. PP-R performed the analysis. XY, PP-R, and BB wrote the manuscript. All authors reviewed the manuscript.

## Conflict of Interest Statement

The authors declare that the research was conducted in the absence of any commercial or financial relationships that could be construed as a potential conflict of interest.
