# Supplementary material for: Identifying Respiration-Related Aliasing Artifacts in the Rodent Resting-State fMRI
Source: Front Neurosci. 2018 Nov 2;12:788. doi: 10.3389/fnins.2018.00788 (PMC6230988; doi:10.3389/fnins.2018.00788)
Supplement: Supplementary file 1 [file Data_Sheet_1.docx]

**Supplementary Figure 1**. **Simulation of the aliasing effect.**


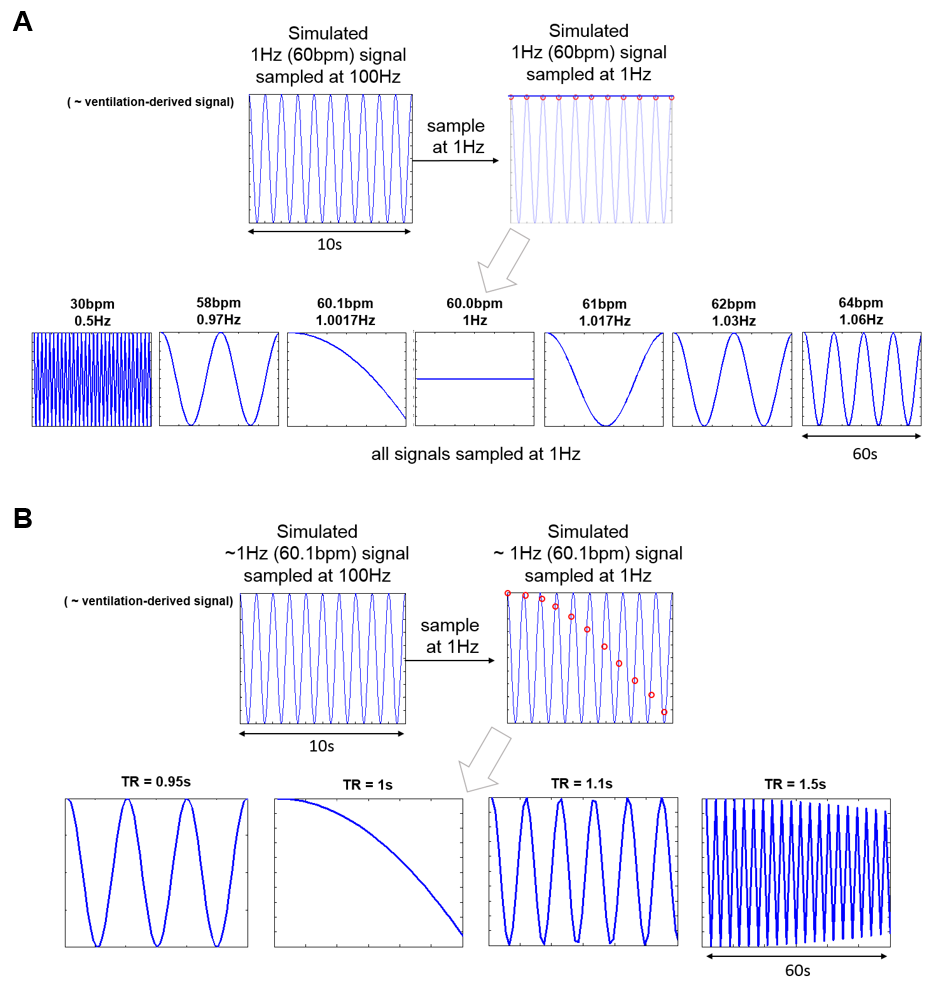


(**A**) The graphs show the signal that would be observed after sampling a periodic movement (e.g. respiratory-driven movement) at 1 s TR. Examples of different ventilatory rates are provided. If the ventilatory rate matches the sampling frequency (1/TR), the resulting signal has frequency = 0, therefore it can be canceled out. By using different ventilatory rates, the frequency of the recorded oscillation varies. (**B**) Effect of the TR on the aliasing of a periodic signal (e.g. respiratory-driven movement) of frequency 1.0017 Hz (60.1 breaths per minute). If the signal was sampled at its exact frequency, a flat line would remain (signal cancellation). By sampling it at a rate close to the ventilatory frequency (sampling the 1.0017 Hz respiratory signal at 1 Hz), a slow wave appears. The waveforms that result from down-sampling the original signal vary their frequency depending on the chosen TR.

**Supplementary Figure 2**. **Aliased ventilation in the fMRI time course observed under different anesthetics.**


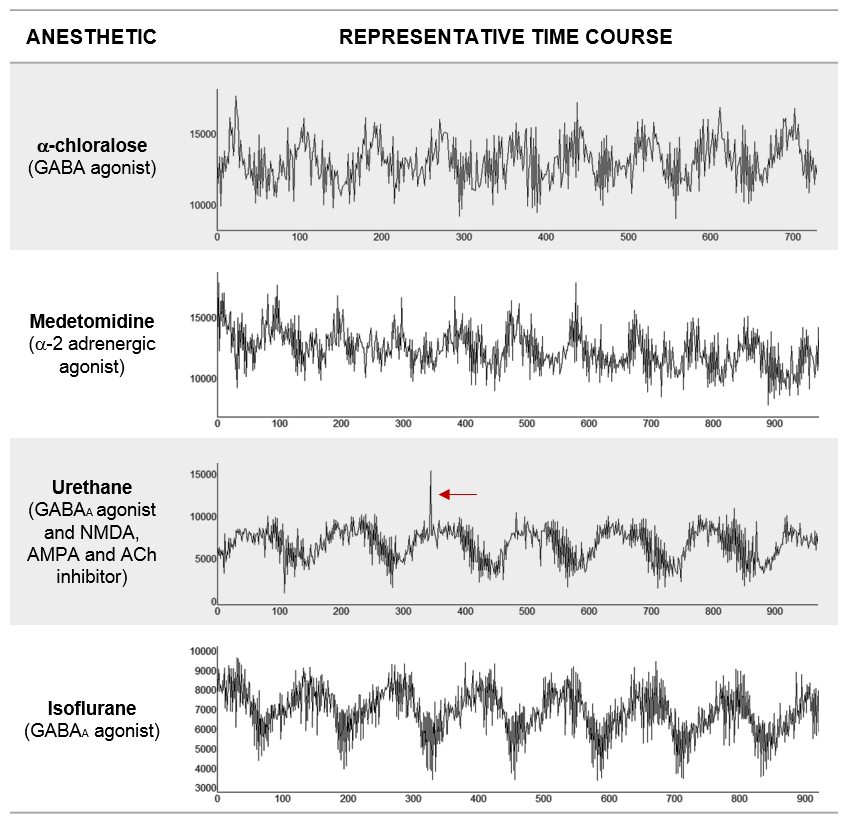


The ultraslow waves can be observed under different anesthetics when the muscle relaxant is not present. The red arrow indicates motion, identified in the time course as a fast change of the BOLD signal, in contrast to the slow aliased signal. TR = 1 s, ventilatory rate near 1 Hz (around 60 bpm).

**Supplementary Figure 3**. **The power map from individual animals.**


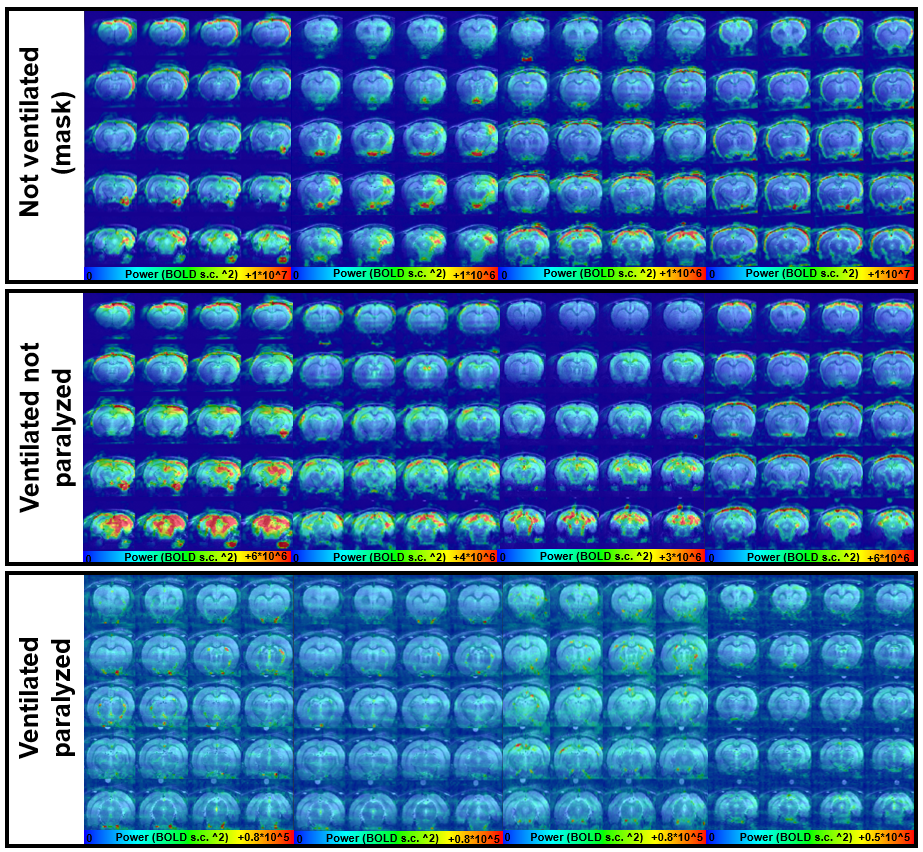


The figure shows 4 individual examples of the power map acquired from animals under 3 different conditions (breathing through a mask -up-, ventilated -middle-, or ventilated and paralyzed -down-). Note how the power of the artifactual oscillations is higher in areas of the periphery, ventral aspect of the brain and occipital lobe/cerebellum, although different patterns can be observed in different animals. When the animal is paralyzed, a more homogeneous pattern is observed, which occurs at a much lower power compared to the non-paralyzed conditions.

**Supplementary Figure 4**.  **Spectral resolution of the aliased respiration assessed with fALFF from mask vs. ventilated non-paralyzed vs. paralyzed animals.**


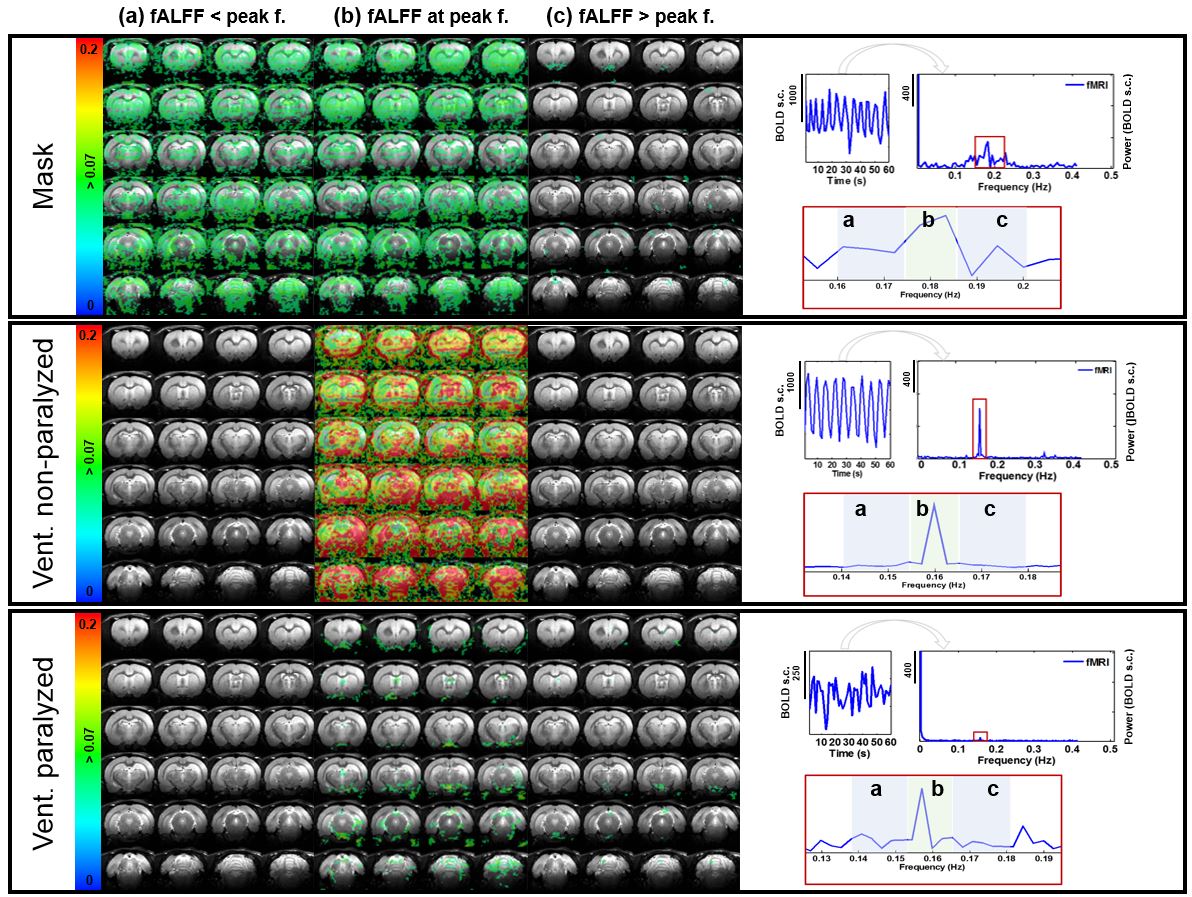


The maps on the left show, for each condition, the fraction of the spectra within the peak frequency range (b), frequency range below the peak (a) and frequency range above the peak (c), with respect to the whole spectrum. On the right, the fMRI time course and PSD of a brain voxel are shown. TR = 1.2 s, ventilatory rate = 1 Hz (60 bpm) in the ventilated conditions.

**Supplementary Figure 5**. **Aliasing of the respiration in the spontaneously breathing animal.**


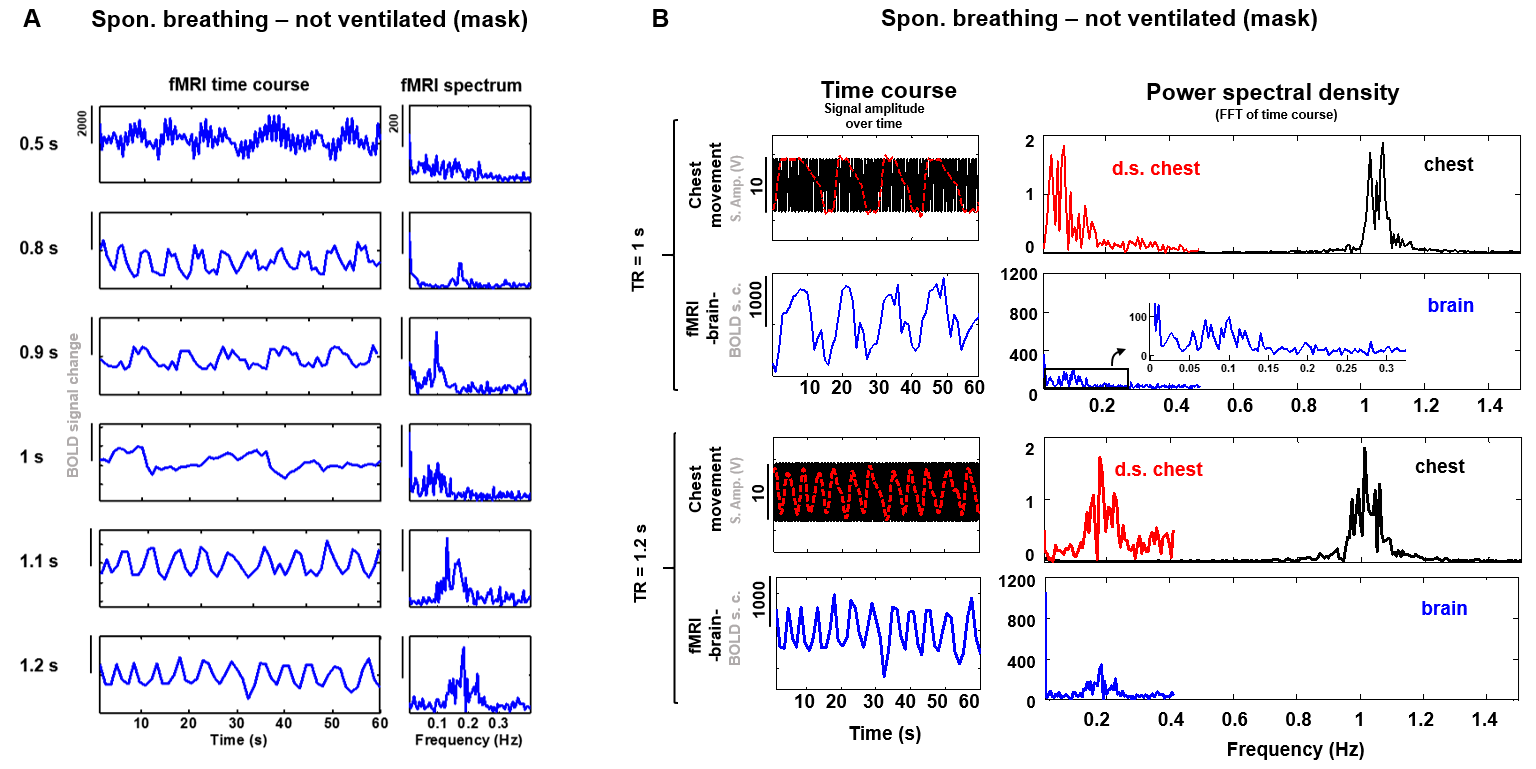


(**A**) The graphs show 1 minute of fMRI time course in a spontaneously breathing non-ventilated animal acquired at 6 different TRs and the corresponding PSDs. (**B**) Two examples showing the chest movement and fMRI signal in time and frequency domain, in an animal breathing through a mask, acquired at TR = 1 s (top graphs) and TR = 1.2 s (bottom graphs). Note how, at 1 s TR, the aliased signal appears within the 0.01 to 0.1 Hz frequency range.

**Supplementary Figure 6**. **Variability of the resting state network identified from non-paralyzed vs. paralyzed animals.**


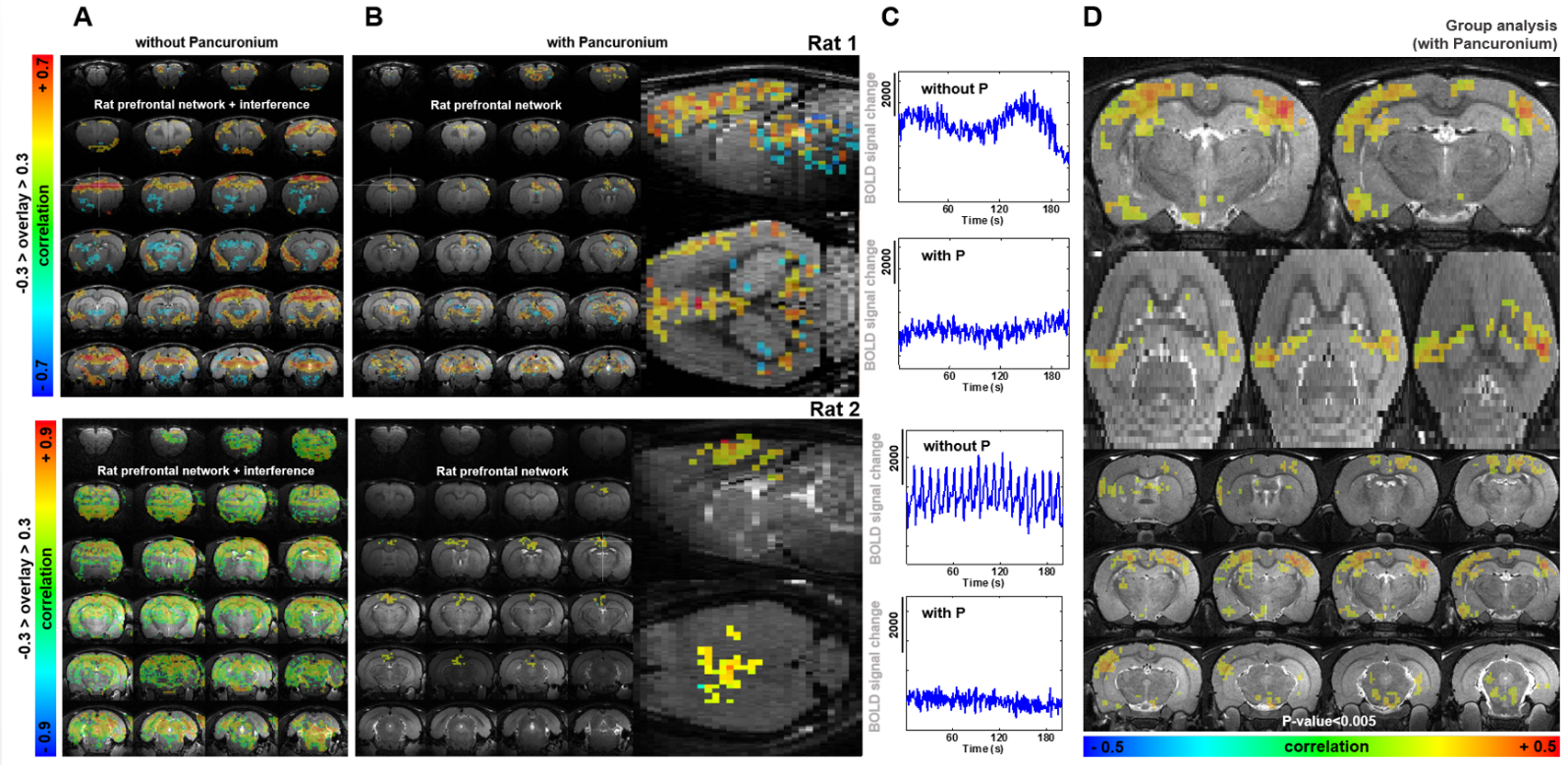


(**A**) Seed-correlation maps acquired from two spontaneously breathing animals ventilated at 60 bpm (TR in Rat 1 = 1 s, and TR in Rat 2 = 1.1 s). (**B**) Seed-correlation map in the same animals after infusion of the muscle relaxant Pancuronium. The cingulate cortex and other prefrontal areas can be clearly identified in the paralyzed animals (**B**) but are overshadowed in spontaneously breathing rats (**A**). (**C**) The graphs show a representative fMRI time course from each rat and condition in A and B. P: Pancuronium. (**D**) Averaged seed-based correlation map from 11 trials in 4 different animals anesthetized with 1.5-2% isoflurane and paralyzed with Pancuronium. Colored voxels exhibit a statistically significant correlation, with *p*-value < 0.005 in a 1 sample *t*-test. Note the clear identification of cortico-cortical connectivity bilaterally.

**Supplementary Figure 7**. **Aliased ventilation in multi-slice (2D) EPI.**


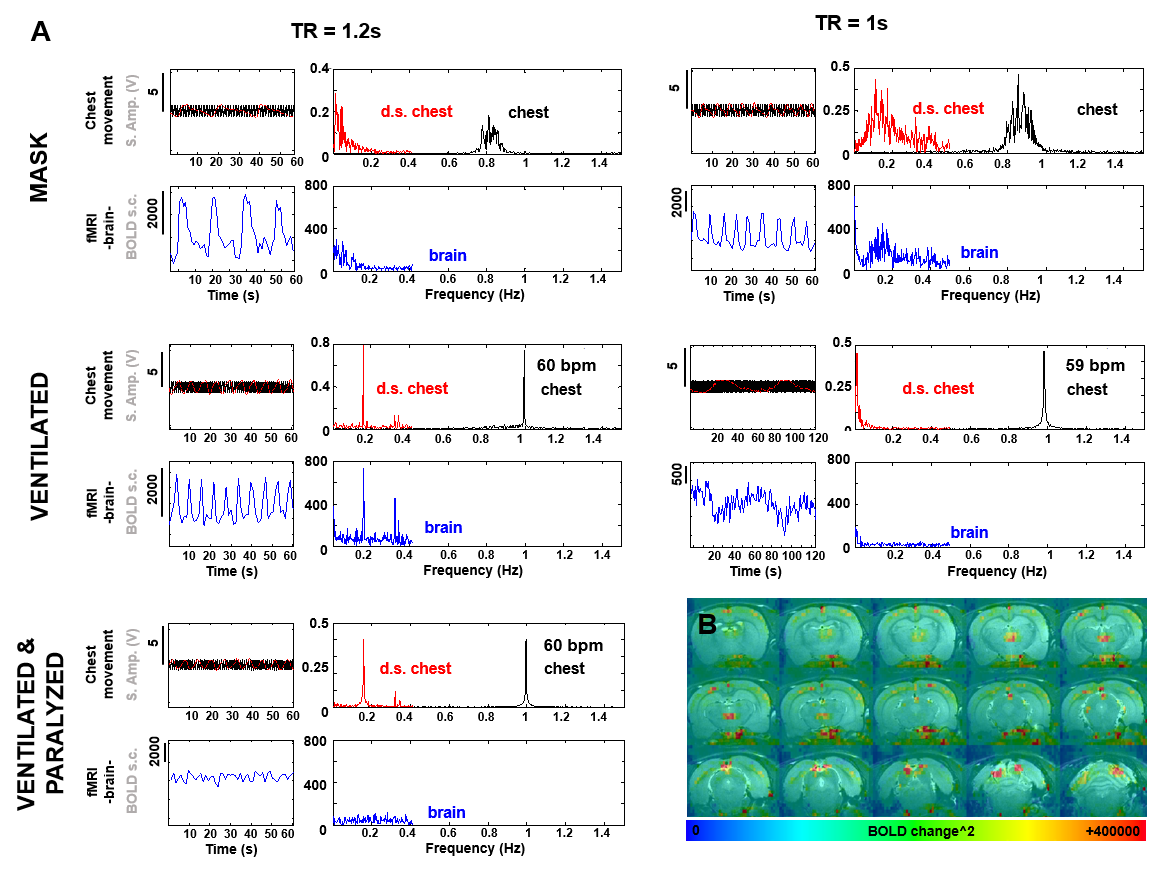


(**A**) The graphs show the time course and PSD of the respiratory chest motion and of the fMRI signal acquired at 2 different TRs in 3 different conditions: animal not ventilated, animal ventilated not paralyzed, and animal ventilated and paralyzed. d.s.: down-sampled to 1/TR. (**B**) The map shows the power distribution of the aliased signal in the ventilated non-paralyzed animal (average of 8 scans from one representative animal). Note the similarity of the aliasing effect between this figure and Fig. 2 (3D EPI), but also the difference in the power maps (the effect is less prominent in 2D EPI).
